# Advancing Surgical Education: A Comprehensive Systematic Review with Meta-Analysis and Novel Approach to Training Models for Local Skin Advancement Flaps

**DOI:** 10.7759/cureus.42066

**Published:** 2023-07-18

**Authors:** Hatan Mortada, Ghaida AlBraithen, Imtinan Al Jabbar, Abdullah Al Qurashi, Nujaim Alnujaim, Saad Alrobaiea, Abdullah E Kattan, Khalid Arab

**Affiliations:** 1 Division of Plastic Surgery, Department of Surgery, King Saud University Medical City, King Saud University, Riyadh, SAU; 2 Department of Plastic Surgery & Burn Unit, King Saud Medical City, Riyadh, SAU; 3 College of Medicine, King Saud University, Riyadh, SAU; 4 College of Medicine, King Khalid University, Abha, SAU; 5 College of Medicine, King Saud Bin Abdulaziz University for Health Sciences, Jeddah, SAU; 6 Plastic Surgery and Burn Unit, Security Forces Hospital, Riyadh, SAU; 7 Division of Plastic Surgery, Department of Surgery, College of Medicine, King Saud University, Riyadh, SAU

**Keywords:** resident training, learning model, advancement flap, local flap, simulation, plastic surgery training

## Abstract

Performing local skin flaps is a challenging task that requires cognitive and technical skills to design flaps with proper orientation to avoid distorting normal anatomy. Junior trainees need adequate exposure to gain confidence and expertise in such procedures. This article systematically reviews the literature's different local skin advancement flap training models and describes a new, easy-to-use training model. A systematic review was performed following the Preferred Reporting Items for Systematic Reviews and Meta-Analyses (PRISMA) guidelines. PubMed, Cochrane, Web of Science, and Google Scholar databases were searched from their inception until August 2022 for articles about local skin advancement flap training models. The meta-analysis results were pooled across the studies using a random-effects model and presented as a weighted mean difference with a 95% confidence interval (95% CI). Out of 773 reviewed articles, 18 were included in the systematic review, and four reported enough data to be included in the meta-analysis. Rhomboid and Z-plasty flaps were the most commonly taught flaps by training models. The most commonly used training models were synthetic-based, followed by animal-based models. The training models significantly increased the trainees' confidence and expertise regarding local skin flap procedures (p<0.00001) for both domains. Training models, per our reported data, significantly improve the trainees' confidence and expertise in performing local skin advancement flap procedures; continuous efforts in developing and establishing new, simple-to-use, and effective training models are strongly encouraged to further improvement of surgical education and enhance the trainees' surgical skills.

## Introduction and background

In recent years, residency surgical training has become more challenging with increased working hours, increasing subspecializations, and centralizing surgical services [1]. The coronavirus disease-19 (COVID-19) pandemic has further complicated practical surgical training due to these factors [2]. As a result, simulation and model-based surgical training have become increasingly popular [3]. 

Through simulation training, trainees can practice procedures efficiently and safely. The training can also provide valuable skills that can be transferred to the clinical setting and positively impact operative outcomes [4]. Local flaps are an essential technique in wound closure because they allow the mobilization of adjacent skin and subcutaneous tissue when direct closure is impossible [4,5]. Flap design and execution is a technically demanding process with cognitive and technical challenges, requiring the design of flaps appropriate to the local anatomy to avoid distortion [6]. It takes extensive exposure and practice for junior trainees to gain confidence and expertise in such procedures [7]. By using models, junior surgeons can practice, refine their skills, and gain confidence in a safe environment to efficiently perform and design local flaps, despite their technically challenging nature.

Many surgical simulators have been mentioned in the literature, but there have yet to be any models for training skin advancement local flaps other than synthetic materials or animals [8]. We aim to highlight all the available models and simulators for teaching local flaps in and to explore their impact on the trainees by conducting a meta-analysis. Furthermore, we describe a simple and affordable training model that trainees from all over the globe could utilize.

## Review

Methods

Systematic Review

This review was conducted by the International Prospective Register of Systematic Reviews (PRISMA) guidelines [9]. The study has been registered in PROSPERO, the International Prospective Register of Systematic Reviews, with the registration number 396851. 

We conducted a systematic literature review using the four major databases, Google Scholar, PubMed, Cochrane, and Web of Science, searching for all the articles discussing the local advancement skin flap training models from the inception of the databases until August 2022. 

The following keywords were utilized: "local advancement skin flaps training", "plastic surgery training models", "cost-effective local flap training models", "advanced flap teaching models", "cadaveric training models", "animal training models", and "computer-based training models". The references of the included articles were reviewed to ensure that all studies were included.

Studies Selection

The systematic review included all articles that discussed training or simulation models for local advancement skin flaps. We ensured quality control by excluding abstracts and letters published only as abstracts and using a reproducible search method. Articles written in languages other than English were not included. We also excluded models not explicitly designed for local flap simulations and articles that did not report relevant outcomes. 

This review excluded articles that examined free flap simulation models. For this systematic review, no limitations were applied regarding publication year, publication status, or type of study. Disagreements regarding selection status were resolved through discussion. The senior author Hatan Mortada (HM) was consulted for a third and final opinion if no consensus could be reached.

Data Extraction

Data extraction was performed by all team members and further checked by the senior author for accuracy. The extracted variables include; country, authors, year of publication, flap type, simulation model, the purpose of developing the simulation model, evaluation method, and advantages and disadvantages, were obtained for the systematic review portion; while the questions assessing the confidence of the trainees before and after utilizing the training models, and the questions assessing their expertise, were extracted to perform the meta-analysis portion. 

Level of Evidence Assessment

We assessed the selected studies for their level of evidence and recommendation using a modified educational Oxford Center for evidence-based medicine categorization system, where a level of recommendation of one is the greatest, and a level of recommendation of four is the lowest [10]. 

Statistical Analysis

All analyses were conducted using RevMan (version 5.4.1; Revman International, Inc., New York City, New York). Means and standard deviations of scoring for the questions assessing the trainee's confidence and expertise levels were extracted from included studies before and after the training models. A weighted mean difference with 95% confidence intervals (CIs) was pooled using a random-effects model. Forest plots were created to evaluate the results of pooling. A p-value less than 0.05 was considered significant; Heterogeneity between trials was assessed using the Higgin I2 test according to the Cochrane Handbook.

Easy-to-Use Training Model

**Figure 1:**
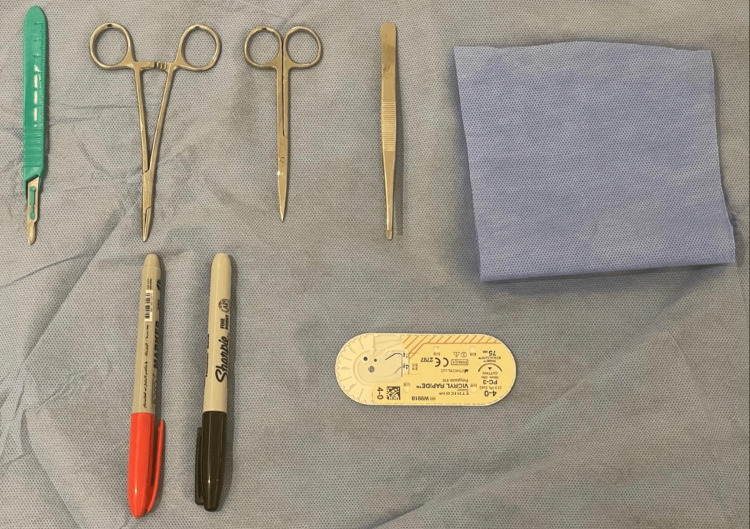
The basic instruments used for training on an easy-to-use model The first author (HM) is the rightful owner of this work.

**Figure 2:**
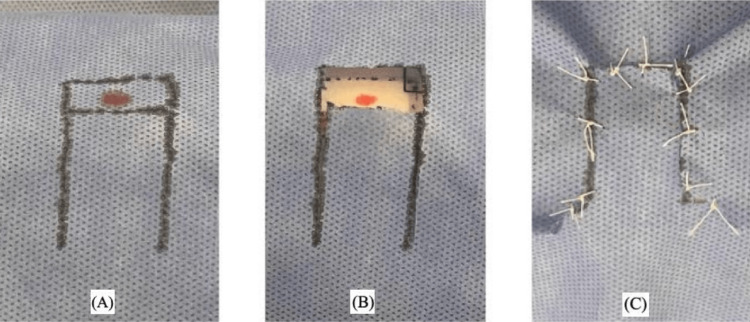
Reversed advancement flap with excision of the Burow's triangle at the base The first author (HM) is the rightful owner of this work.

**Figure 3:**
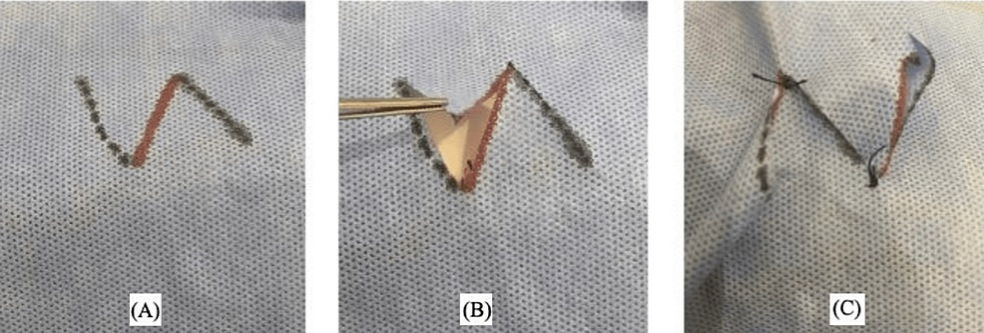
Z-plasty flap The first author (HM) is the rightful owner of this work.

**Figure 4:**
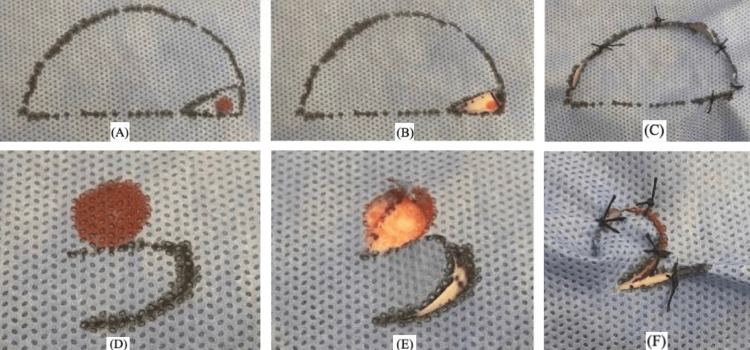
(A-C) classic rotational flap showing the pivot point; (D-F) circular defect covered by a local flap The first author (HM) is the rightful owner of this work.

We present five uses of this model in different local skin advancement flaps. The primary instruments needed for applying this training model are a piece of a surgical drape, forceps, suture forceps, scissors, blades, markers, and needle holders (Figure 1). Any suture from 1-0 to 4-0 can be used for the suturing. With a thickness of 1-2 mm, a piece of a surgical drape (5x5 cm) was cut to size to fit into the hanger. Following the marking of the flap design with a marker, the blade was used to cut through the cloth, and the incision was rotated to aim at the flap, as shown in Figure 2. Using sutures, the flap is rotated and fixed accordingly (Figures 2-4).

Results

Systematic Review 

Of 773 identified studies, 18 articles met our inclusion/exclusion criteria [3,7,11-26] and were included in the meta-analysis portion. Four studies reported data about the trainee's confidence following the utilization of the training models, and two studies reported data about the expertise level of the trainees after and before using the training models. All studies included in this review were published between 2003 and 2021. 

**Figure 5:**
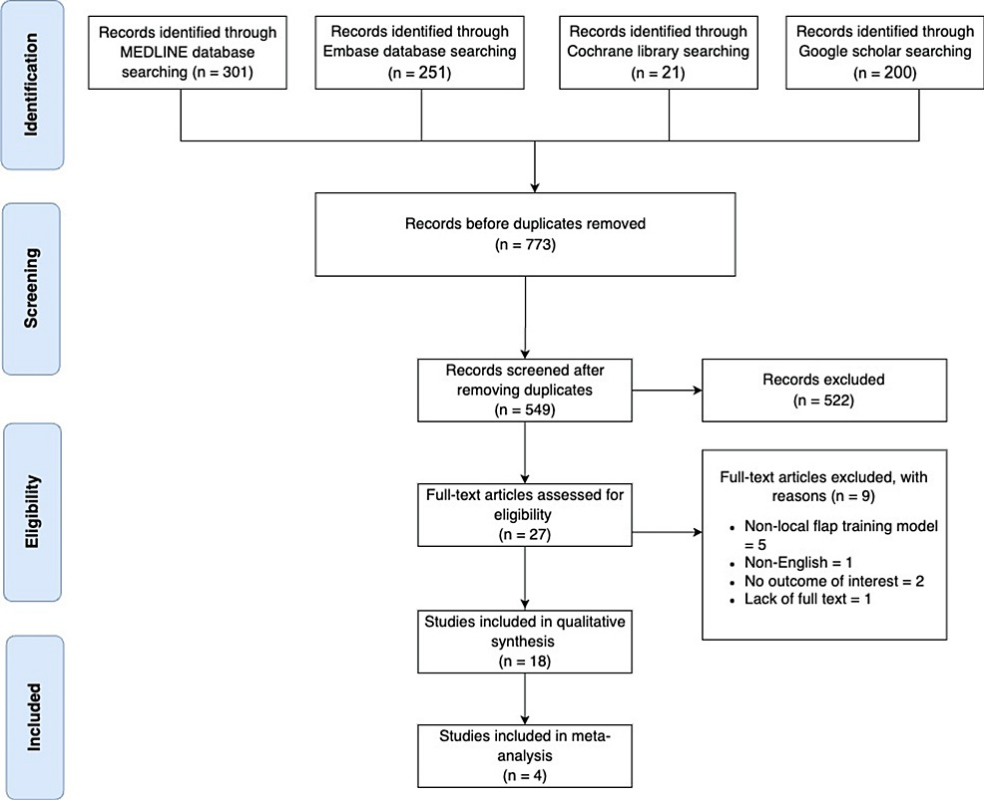
PRISMA chart used for conducting the systematic review

Among all the studies included, eight articles discussed the study design. Two of the studies were randomized controlled trials [15,25], four were cross-sectional studies [7,13,16,17], one was a prospective cohort study [3], and one was a review article [12]. The studies were conducted in different parts of the world, including seven articles in the United States of America (USA), three articles in the United Kingdom (UK), three articles in Germany, and one article in Turkey, Sweden, Malaysia, and Japan. Figure 5 summarizes the PRISMA methodology for conducting a systematic review.

Study Characteristics 

Across all studies, 324 candidates participated in different types of flap training. Four articles did not specify the number of candidates. The level of training and specialty of the candidates included in the different studies were diverse and ranged from medical students to faculty members. In total, four articles focused on medical students (n = 4), four articles on plastic surgery residents (n = 4), four articles on ear, nose, and throat (ENT) residents (n = 4), one article on dermatology residents (n = 1), and two articles mentioned surgical residents. 

The three most frequently mentioned flaps were the rhomboid flap (mentioned four times), the Z-plasty (mentioned four times), and the Bilobed flap (mentioned three times). These flaps are the most commonly taught in surgical training programs. Table 1 highlights the characteristics of the included studies. 

**Table 1:** Basic characteristics of studies included in the review USA - United States of America, UK - United Kingdom, NM - not mentioned, ENT - ear, nose, and throat.

Authors	Journal	Study Design	Country	Sample Size	Level and specialty of the candidates	Cost	Evidence
Ederer (2021) [7]	Journal of Surgical Research	Cross-sectional	Germany	9	First year (4), third year (1), fourth year (2), and sixth year (2) plastic surgery residents	Cost-effective	3
Kwan (2020) [11]	International Journal for Numerical Methods in Biomedical Engineering	NM	Malaysia	NM	NM	NM	
Chan (2010) [12]	Journal of Plastic, Reconstructive & Aesthetic Surgery	Review article	UK	NM	NM	£2.79	3
Khalil (2009) [13]	Dermatologic Surgery	Cross-sectional	Germany	71	Surgical residents	NM	3
Yang (2021) [3]	The Laryngoscope	Prospective cohort	USA	15	ENT residents from all years	NM	2b
Powell (2019) [14]	JAMA Facial Plastic Surgery	NM	USA	7	ENT-trained facial plastic surgeons	Small facial flap $4.61, large facial flap $8.41	3
Naveed (2018) [15]	Advances in Simulation	Randomized educational trial	UK	20	Medical students	NM	2b
Kite (2018) [16]	Plastic and Reconstructive Surgery - Global Open	Cross-sectional	USA	18	Nine plastics surgery residents from one to five years two faculty attending surgeons General surgery-trained burn fellow Plastic surgery nurse practitioner	Flat LFT $150, 3D face model $200	3
Ueda (2017) [17]	Plastic and Reconstructive Surgery	Cross-sectional	Japan	6	Plastic surgery residents	Cost 30-60 USD	4
Taylor (2016) [18]	SAGE- Otolaryngology-Head and Neck Surgery	NM	USA	10	ENT residents and faculty	Cost-effective	3
Mitchell (2015) [19]	Plastic and Reconstructive Surgery	NM	USA	18	Residents, clinical and research fellows, plastic surgery-aculty	NM	
Bauer (2015) [20]	Head & Face Medicine	NM	Germany	19	Medical students	NM	3
Isaacson (2014) [21]	The Laryngoscope	NM	USA	16	ENT residents, physicians, students	Cost-effective	3
Hassan (2014) [22]	Annals of Plastic Surgery	NM	UK	NM	NM	NM	4
Denadai (2014) [23]	The Journal of Craniofacial Surgery	Randomized controlled trial	Brazil	60	First and second year medical students	Cost-effective	2b
Sifakis (2009) [24]	Studies in Health Technology and Informatics	NM	USA	NM	NM	NM	4
Bjellerup (2005) [25]	American Society for Dermatologic Surgery	NM	Sweden	22	Dermatology residents	NM	3
Altinyazar (2003) [26]	American Society for Dermatologic Surgery	NM	Turkey	33	Interns and first year residents	Cost-effective	3

Overview of Local Flap Training Models

In this study, we reviewed 18 training models for local flaps. Six of these were animal-based models, using cadaveric cattle digits, chicken thigh, pig head, porcine, rat's skin, and turkey thigh. Ten models were synthetic based, with four of them being 3D models and the rest utilizing materials such as Allevyn dressing (Smith & Nephew, London, United Kingdom), gelatin skin, or didactic materials. One study employed the use of human skin, and four articles focused on computer-assisted and virtual reality simulation, including mobile simulation applications or computer-based systems. 

Regarding the evaluation methods, a variety of techniques were used. However, the most prevalent was the questionnaire-based evaluation method. Despite the diversity in the evaluation and progress assessment methods, most studies demonstrated a positive impact of the training models and a marked improvement in the participants' skills. Table 2 summarizes the advantages and disadvantages of each of the training models reviewed in this review. The costs of the training models were mentioned in five articles, while several other articles only mentioned "cost-effective" without specifying the exact amount. Costs ranged from 3.83 USD to 350 USD. Table 3 summarizes the advantages and disadvantages of various local flap training models and clinical recommendations. 

**Table 2:** Variables related to flap training models and evaluation method NM - not mentioned, OSATS - Objective Structured Assessment of Technical Skills Score, RFF - radial forearm flap, ND - neck dissection, FTSG - full thickness skin graft, SSG - split thickness graft

Authors	Type of flap training	Model type (Synthetic, animal)	Tissue or material	Evaluation method
Ederer (2021) [7]	Transposition ﬂap, rotation ﬂap, advancement ﬂap, and Z-plasty.	Human skin	Fresh human skin excised from body contouring procedures.	Questionnaire according to the modiﬁed version of the Objective Structured Assessment of Technical Skills Score
Kwan (2020) [11]	Advancement flap, rotation flap, rhomboid flap	Synthetic (3D model)	3D Asian head templates of two opposite genders by using finite element method	Observing the results on the model
Chan (2010) [12]	Different local flap techniques including advancement flap	Animal - chicken thigh	Chicken thigh	NM
Khalil (2009) [13]	Z-plasty, V-Y plasty, and oval-shaped advancement flap	Animal - cadaveric cattle digits	Cadaveric cattle digits	Questionnaire - Likert scale ranging from 1 to 6, handed to the residents to evaluate the 75-minutes skills course
Yang (2021) [3]	Rhomboid flap, O-T flap	Synthetic (3D model)	3D printed facial ﬂap simulator	1. An evaluation survey based on a Likert scale 2. An experienced facial plastic and reconstructive surgeon rates the surgical skill of each candidate blinded
Powell (2019) [14]	Z-plasty, V-Y plasty, rhombic flap, rectangular advancement, bilobe, O-T, pinwheel	Computer and virtual simulation	Computer-aided 3D simulator	Questionnaire survey based likert scale
Naveed (2018) [15]	Elliptical closure, bilateral advancement (H flap) and the semicircular rotation flap	Computer and virtual simulation	Mobile simulation app - BaSSis mobile app	A plastic surgeon blinded to the allocation participants' multiple-choice questions rated the OSATS task-based assessment
Kite (2018) [16]	Rhomboid flap bilobed rotational 2-plasty bilobed nasolabial forehead flap	Synthetic - local flap trainer (LFT) consisted of a foam core base overlaid with multiple silicone layers over a 3D head and neck bust LFT	3D simulator	Questionnaire survey using a 1-10 scale
Ueda (2017) [17]	Bilobed flap and cheiloplasty	Synthetic - made of outlayer of polyurethane and inner layer of silicone	3D - three-dimensional computer-assisted two-layer elastic models of the face and cleft lip from the computed tomographic and magnetic resonance imaging stereolithographic data. The surface layer is made of polyurethane and the inner layer is silicone.	"Enjoyable and realistic experience" "Can understand 3D movement of the flap"
Taylor (2016) [18]	Bilobed flap, paramedian forehead, rhombic, z-plasty	Synthetic	Gelatin skin	Questionnaire survey based Likert scale
Mitchell (2015) [19]	Z-plasty, rhombic, S-plasty, rotational flap dufour-mentel mouly	Computer and virtual simulation	Computer-based	"Responses were recorded in prose for use in simulator updating."
Bauer (2015) [20]	RFF, ND, FTSG, SSG, U flap, rotational flap, bilobed, Z-plasty	Animal	Pig head	Written test followed by Likert scale questionnaire
Isaacson (2014) [21]	Rhomboid, bilobed, rotation island transposition flaps, Z-plasty	Animal	Turkey thigh	Questionnaire survey
Hassan (2014) [22]	Transposition, rotation, rhomboid, square peg in a round hole flap, forehead, glabella, bilobed nasolabial hatchet, Abbe-Estlander McGregor cheek flap	Animal	Porcine skin	Feedback
Denadai (2014) [23]	Rhomboid flaps	Synthetic and animal	Didactic materials, rubber, ethylene-vinyl acetate, chicken leg skin, pig foot skin	Recording, questionnaire-based Likert scale, global rating scale
Sifakis (2009) [24]	Local flaps	Computer and virtual simulation	Computer simulation	5-point scale
Bjellerup (2005) [25]	A-T plasty, H-plasty, U-plasty, rotational flap	Synthetic	Allevyn dressing (non-adhesivehydrocellular polyurethane dressing)	1-5 scale
Altinyazar (2003) [26]	Rotational and Z-plasty	Animal	Rats' skin post-experimental studies	1-5 scale

**Table 3:** A review of the literature regarding the advantages and disadvantages of different local flap training models and clinical recommendations NM - not mentioned, RCT - randomized controlled trial

Authors	Advantages	Disadvantages	Clinical recommendations
Ederer (2021) [7]	Adequate model for medical students to practice	Selection bias	Hands-on courses for complex defects demonstrate an excellent way to recruit more surgeons and build on their skills.
Kwan (2020) [11]	Resemble real skin and its qualities, good for training in the art of flap design and execution	Controversial, hard to use in the practice of flaps around complex facial angles and ridges	Porcine skin is a realistic teaching model that was proven satisfactory among our population, and closely resembles cadaveric tissue without the ethical dilemma.
Chan (2010) [12]	Usable, cheap, available, excellent media for practice	Inability to practice deep sutures	The close resemblance to human skin and the ease of setup make this teaching tool optimal for trainees to acquire simple surgical skills.
Khalil (2009) [13]	Noninfectious, skin layer thickness measured almost identical to human, size permits the practice of multiple procedures, elasticity almost that of human skin permitting movement.	Essential skin layer is lost due to the defeathering process making it an unsuitable model for facial reconstruction procedures requiring a thick layer of dermis and subcutaneous fat.	Novel galliform model using fresh turkey thighs skin components almost histologically identical to human facial skin making it a good tool for flap and suture practice especially for facial plastic surgery.
Yang (2021) [3]	1. High-fidelity skin properties, including rigidity and elasticity 2. Huge skin area that allows training on several wound closures 3. No ethical concerns	1. Lack of comparison between the training model used in this study and other models 2. The model has to be prepared before use.	It is recommended that young trainees attend skills training courses to learn advanced wound closure techniques. The curriculum based on cattle digits presented here is recommended.
Powell (2019) [14]	NM	NM	This simple model has been used to develop a system for the practice of common plastic surgical skills in a non-clinical setting, which has aided in the training of junior doctors in the department. Their immediate trainers can observe their skills in order to assess their competency prior to undergoing 'live surgery'. This practical and cost-effective skills training is highly recommended.
Naveed (2018) [15]	1. High-fidelity skin properties and anatomical structure allows an accurate differentiation of the skin layers 2. Can be frozen and stored to be used later, yet still providing fresh human skin features	1. Ignoring the anatomical landmarks for a location-based approach of flap-specific training 2. Small sample size 3. Trained residents are of different levels	It has been demonstrated that the simulation model based on fresh human skin is cost-effective and can be applied to a broad range of flap procedures, which is why its use should be further promoted.
Kite (2018) [16]	1. By adding tension to the springs between the epidermis layers, we can achieve a more realistic effect of wound opening during cutting	1. It was not possible to ensure that participants did not read more about skin surgery and local flaps outside of the course 2. Sample size is small	Through touch-based simulation, three different flap techniques for facial soft tissue reconstruction were learned efficiently and effectively, and steps involved in the surgery were recalled in a fluid manner that improved task performance.
Ueda (2017) [17]	Properties similar to that of real skin, sutures remain in place without assistance, trainees can keep their simulator for further practice	NM	Allevyn is superior to its alternatives for teaching and mastering various skin flaps, e.g. pig skin, as it better resembles human skin and its mechanics resulting in optimal flap training conditions highly rated by trainees.
Taylor (2016) [18]	1. Accurate surgical simulation of both anatomy and tools. 2. Enables the development of new approaches with precision	NM	This local flap training model proved to be an excellent tool to be utilized by plastic surgeons and has shown the potential for use in teaching both current procedures and implementing new and uniquely designed approaches for unique injuries, e.g. shrapnel and war injuries.
Mitchell (2015) [19]	Allows trainees to practice without harm to patients, resembles real-life patients, helps in cases of patient-specific procedures/approaches	Graphics are needed to indicate areas of stress in secondary closure, recording, indicators, instructor grease pencils, more detailed microanatomy	Our preliminary flap simulator has proven to be a successful educational platform for local flap practice. Future work includes adapting the system to other surgical areas.
Bauer (2015) [20]	Multi-use, simply made, resembles real facial skin facilitating multiple flaps	Evaluation sample is not enough, missing tissue elasticity element	The gelatin facial flap gives doctors the opportunity to safely learn and conduct facial flaps due to adequately simulating human tissue although further investigation is needed.
Isaacson (2014) [21]	Replicates elasticity of natural skin	NM	Our suture pad simulates human tissue well. In flap workshops, this suture pad is a valuable teaching tool.
Hassan (2014) [22]	Enables an understanding of 3D design and flap movement simulates the operation of face-like structures that have complicated 3D structures	NM	Using this elastic model, we taught residents and young doctors how to make several typical local flaps and to perform cheiloplasty. They could experience realistic simulated surgery and understand three-dimensional movement of the flaps.
Denadai (2014) [23]	A thorough simulation of the advancement, rotation, and rhomboid flaps closure on a realistic one-layered 3D model of both genders	1. Ignoring the multiple layering and pre-stress effects 2. Apart from the 3D Asian head templates, the rest of the skin thickness parameters were gathered from cadaveric Caucasian population, even though the study was meant to investigate the Asian population N/A	Predicting the biomechanics and the wound closure of Advancement, rotation, and rhomboid flap designs on both genders
Sifakis (2009) [24]	1. The use of simulation as a realistic training tool 2. Assessment of trainee competency using the preferred model 3. Training tool for COVID-19 and scenarios requiring a reduction in traditional healthcare operations	NM	In the surgical education of facial reconstruction, trainees found the facial flap simulator to be an effective and useful training tool with a high level of realism. In extraordinary circumstances, such as the novel Coronavirus disease pandemic of 2019, surgical simulators can provide a valuable addition to training, especially during times of high need.
Bjellerup (2005) [25]	1. Realistic and anatomical accuracy 2. Great potential for learning local facial flaps 3. Reusability 4. Ability to practice on the anatomical landmarks of facial features	Requires a thinner skin layer, increased elasticity, and a softer adipose layer	Realism, experience, performance, and usefulness of the local facial flap simulator were highly rated by experts. If refined slightly, the model can be used widely in otolaryngology–head and neck surgery and facial plastic surgery training
Altinyazar (2003) [26]	Recordable and blinded unbiased assessment, provides performance analysis along multiple areas, increase in flap training and better execution, recyclable, adaptable, available, provide feedback.	Limited to rhomboid flaps, doesn't provide information on flap retention, only evaluated on one small sample of medical students from the same center.	This RCT showed that medical students mastered rhomboid flap regardless of the level of fidelity.

Meta-analysis Results

**Figure 6:**
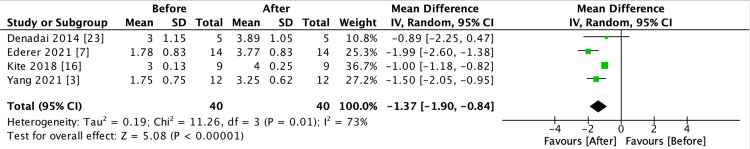
Forest plot of the trainees' confidence level before and after utilizing the training models

Trainee's confidence before and after utilizing training models: Yang et al., Ederer et al., Kite et al., and Denadai et al., reported data about the trainees' confidence in performing local skin flaps before and after utilizing the training models [3,7,16,24]. We pooled the mean score and the standard deviation of the participants in their study to investigate the impact of such training models on the trainees' confidence and found that utilizing a training model significantly increases the trainee's confidence (p<0.00001). Further details are depicted in the forest plot, Figure 6.

**Figure 7:**
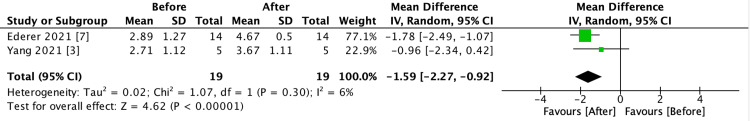
Forest plot of the trainees' expertise level before and after utilizing the training models.

Trainee's expertise before and after utilizing training models: Yang et al. and Ederer et al. reported data about the trainees' expertise in performing local skin flaps before and after utilizing the training models [3,7]. We found that utilizing a training model significantly increases the trainee's expertise regarding local skin flaps (p<0.00001). Further details are depicted in the forest plot, Figure 7.

Quality Assessment and Risk of Bias 

Following the Oxford Center for evidence-based medicine's modified classification system, where a level of recommendation of 1 represents the highest level of recommendation, and a level of recommendation of 4 represents the lowest level of recommendation [10], we graded the selected studies in terms of their level of evidence and recommendation. Ten articles were rated level 3, three were rated level 4, three were rated level 2b, and two could not be assessed due to insufficient data (Table 1).

Discussion

Training on local flap techniques is essential for residents in plastic and reconstructive surgery [3]. These procedures are often complex and require a thorough understanding of anatomy, blood supply, and surgical technique. It is expected that residents who have been trained in local flap techniques will be better able to handle a variety of reconstructive cases and will be able to provide their patients with more effective care [8]. This, in turn, can lead to improved patient outcomes and a better overall experience for the patient. 

Various obstacles can prevent new surgeons from learning and acquiring surgical skills, ranging from the challenging and stressful operating theatre environment to patient safety concerns and increased working hours, which limit the time available for bedside teaching [9]. Here is where the surgical training models come in handy. Even though these models cannot replace real clinical experience and bedside teaching, they facilitate the learning process. This article aims to provide a comprehensive review of flap simulation methods available and their impact to surgical trainees, as well as highlighting our skin advancement local flap training model, which is easy to use, accessible, and readily available. Our systematic review and meta-analysis included 773 articles across different databases; only 18 were included in the systematic review, and four were included in the meta-analysis, with 324 candidates participating in different flap training models. 

Rhomboid and Z-plasty flaps were the most commonly taught flaps by those training models. The most commonly used training models were synthetic-based, followed by animal-based models. 

In the literature, various training models have been described for teaching local flaps, including fresh human skin, animal skin, cadaveric skin, and 3D simulators [8]. This emphasizes the importance of involving simulation models in the teaching process. However, not all of these models are readily accessible to surgical trainees and junior surgeons. For example, plenty of abdominoplasties should be performed to provide adequate fresh human skin for training [7]. Conversely, cadaveric skin and 3D simulators are costly. Recently, various ethical issues have arisen regarding the use of animal skin for training [8]. 

Artificial intelligence (AI) has been increasingly employed in various aspects of medicine, including surgical training [27]. Although in its nascent stages, AI-based models hold significant potential in the domain of reconstructive surgery, especially in flap raising and insetting training [28]. Machine learning and deep learning algorithms, capable of learning from complex and extensive datasets, could assist in crafting realistic surgical simulations and providing objective, real-time feedback to residents [29]. However, literature on this specific application of AI in the local skin advancement flap training model is limited.

Incorporating AI into surgical education could revolutionize training approaches, creating a safer, more effective learning environment [29]. Advanced simulations could offer residents hands-on experience with flap design, raising, and insetting and tailor learning to individual skill levels. Yet, despite these promising prospects, there is a clear need for additional research to validate the effectiveness and feasibility of these models in day-to-day surgical training.

In this study, we shed light on a home-made, simple-to-use, easy-to-handle, and time-saving training model by which plastic surgery residents can improve their surgical skills in performing different types of local flaps, including classic unidirectional advancement flaps, V-Y advancement flaps, Z-plasty, rotational flaps, and bilobed flaps. The primary material used to create this model is easily accessible to any household. In addition, to surgical instruments that any surgical resident of any other discipline would causally own. The time required to build this model is only one to two minutes. However, our model has its limitations. The first problem is that this model needs more realism regarding elasticity and rigidity. Secondly, it lacks the multiple layers of natural skin. Several of the models described in this review are also at an early stage of development. These have been included because they are undoubtedly exciting and significant when indicating potential future directions for simulation training in local flap surgery. This article clearly illustrates the usefulness of this model despite its limitations. Our recommendation for future articles is to assess whether junior surgeons are satisfied with this training model and if any improvements can be made. This innovative, simple-to-use model meets most surgical residents' needs. However, further studies need to observe how plastic surgery residents, in particular, and surgical residents from other disciplines, in general, improve after completing skill courses using this model.

## Conclusions

In our systematic analysis, most models described were evaluated in only a few cohorts. As a result, larger candidate sizes will be required, as well as standard assessment methods. The meta-analysis showed a significant improvement in the trainees' expertise and confidence levels following the training models' usage.

We also proposed a new model to be utilized by the trainees. An improvement in training in a complex area of surgery such as this will require further development and evaluation of promising high-fidelity models. A continuous effort in building and establishing innovative training models is strongly encouraged to improve further the trainees' confidence in performing complex procedures.
